# Nomogram Models to Predict Postoperative Hyperlactatemia in Patients Undergoing Elective Cardiac Surgery

**DOI:** 10.3389/fmed.2021.763931

**Published:** 2021-12-02

**Authors:** Dashuai Wang, Su Wang, Jia Wu, Sheng Le, Fei Xie, Ximei Li, Hongfei Wang, Xiaofan Huang, Xinling Du, Anchen Zhang

**Affiliations:** ^1^Department of Cardiovascular Surgery, Union Hospital, Tongji Medical College, Huazhong University of Science and Technology, Wuhan, China; ^2^Department of Emergency Medicine, Union Hospital, Tongji Medical College, Huazhong University of Science and Technology, Wuhan, China; ^3^Key Laboratory for Molecular Diagnosis of Hubei Province, The Central Hospital of Wuhan, Tongji Medical College, Huazhong University of Science and Technology, Wuhan, China; ^4^Department of Cardiovascular Surgery, First Affiliated Hospital of Zhengzhou University, Zhengzhou, China; ^5^Department of Nursing, Huaihe Hospital of Henan University, Kaifeng, China; ^6^Department of Cardiology, The Central Hospital of Wuhan, Tongji Medical College, Huazhong University of Science and Technology, Wuhan, China

**Keywords:** cardiac surgery, postoperative hyperlactatemia, prediction model, nomogram, risk factor

## Abstract

**Objectives:** Postoperative hyperlactatemia (POHL) is common in patients undergoing cardiac surgery and is associated with poor outcomes. The purpose of this study was to develop and validate two predictive models for POHL in patients undergoing elective cardiac surgery (ECS).

**Methods:** We conducted a multicenter retrospective study enrolling 13,454 adult patients who underwent ECS. All patients involved in the analysis were randomly assigned to a training set and a validation set. Univariate and multivariate analyses were performed to identify risk factors for POHL in the training cohort. Based on these independent predictors, the nomograms were constructed to predict the probability of POHL and were validated in the validation cohort.

**Results:** A total of 1,430 patients (10.6%) developed POHL after ECS. Age, preoperative left ventricular ejection fraction, renal insufficiency, cardiac surgery history, intraoperative red blood cell transfusion, and cardiopulmonary bypass time were independent predictors and were used to construct a full nomogram. The second nomogram was constructed comprising only the preoperative factors. Both models showed good predictive ability, calibration, and clinical utility. According to the predicted probabilities, four risk groups were defined as very low risk (<0.05), low risk (0.05–0.1), medium risk (0.1–0.3), and high risk groups (>0.3), corresponding to scores of ≤ 180 points, 181–202 points, 203–239 points, and >239 points on the full nomogram, respectively.

**Conclusions:** We developed and validated two nomogram models to predict POHL in patients undergoing ECS. The nomograms may have clinical utility in risk estimation, risk stratification, and targeted interventions.

## Introduction

Postoperative hyperlactatemia (POHL) is a common metabolic disorder in patients undergoing cardiac surgery and is associated with adverse clinical outcomes, such as severe acute kidney injury, acute respiratory distress syndrome, and mortality (1–3). Given that elevated lactate levels have important implications for poor prognosis, lactate levels could represent a useful goal of initial resuscitation (4). Nonetheless, studies on lactate-directed therapy performed in post-cardiac surgery patients are limited (5). The reported incidence of POHL in different studies varied greatly, from 11.1 to 45.6% (6, 7).

Globally, numerous studies have been conducted to explore predictors for POHL after cardiac surgery due to its high prevalence and poor outcomes (8–10). Some significant risk factors have been reported in the literature, such as non-elective surgery and diabetes mellitus (8, 11). However, the baseline characteristics of the surgical patients have changed substantially these years due to great advances in anesthesia and surgical techniques. Many studies were published decades ago, and some conclusions may have been obsolete because of the very limited samples and narrow patient selection (3, 12). In addition, the majority of the published studies were conducted in patients undergoing mixed surgeries or single valvular heart surgery (13, 14), but none were conducted specifically for patients undergoing elective cardiac surgery (ECS). To our knowledge, a large-scale persuasive study on this topic is still lacking, and the establishment of a convincing prediction model is still an urgent need.

The aim of this study was to identify independent risk factors for the development of POHL in adult patients who underwent ECS and to develop and validate two easy-to-use clinical prediction models that may help clinicians evaluate the risk of POHL and implement appropriate interventions early.

## Methods

### Ethical Statement

This study was conducted according to the ethical statement of the Declaration of Helsinki and was approved by the ethical committee of the Union Hospital (No. 0521), Tongji Medical College, Huazhong University of Science and Technology, Wuhan, China. Individual consent was waived due to the retrospective nature.

### Study Design and Patient Population

We conducted a multicenter retrospective study consisting of all the adult patients (age ≥ 18 years) who were admitted to the intensive care unit (ICU) after ECS, at four tertiary care academic centers for a period of 5 years from January 2016 to December 2020. Exclusion criteria were patients who experienced emergency cardiac surgery, organ transplantation, immunosuppression or immune deficiency, and intraoperative death.

The following information was collected: age, sex, body mass index, drinking history, smoking history, hypertension, diabetes mellitus, chronic obstructive pulmonary disease, general surgery history, cardiac surgery history, peripheral vascular disease, cerebrovascular disease, gastrointestinal tract disease, renal insufficiency, atrial fibrillation, pericardial effusion, left ventricular ejection fraction (LVEF), New York Heart Association class, pulmonary artery hypertension; white blood cell count, red blood cell (RBC) count, hemoglobin, serum albumin, globulin, and creatinine; aortic cross-clamp time, cardiopulmonary bypass time, and intraoperative transfusion of RBC.

### Outcomes

The primary outcome was the occurrence of POHL in patients undergoing ECS. The secondary outcomes were readmission to ICU, in-hospital mortality, the length of ICU, and hospital stay.

### Measurement and Definition

Lactate was routinely measured from ICU admission to ICU discharge in these hospitals. Based on clinical practice and previous literature, POHL was defined as lactate level >4 mmol/L in this study (15, 16). All the available lactate values within the first 12 h after ICU admission were recorded, and the peak values were used to identify the presence or absence of POHL.

### Statistical Analysis

Descriptive statistics were used to summarize baseline data of the study population. Continuous variables were presented as means with standard deviations when normally distributed and medians with interquartile ranges when non-normally distributed. Categorical variables were expressed as frequencies with percentages. Missing values were imputed by applying multiple imputations. We first performed a univariate analysis to screen possible risk factors. Student's *t*-test, a non-parametric test, Chi-square test, and Fisher's exact test were used as appropriate. Variables with *p* < 0.1 were further analyzed to identify independent predictors by forwarding stepwise multivariate logistic regression. Results from logistic regression analysis were expressed as odds ratios (ORs) with 95% CIs. A nomogram based on the multivariate model was then established.

Internal validation of the nomogram model was evaluated by bootstrapping using 1,000 replications. External validation was performed in an independent validation cohort. To assess the calibration of the model, a smoothed non-parametric calibration curve and a fitted logistic calibration curve were depicted. To evaluate the discrimination of the model, the area under the receiver operating characteristic (ROC) curve (AUC) or the c-index was calculated. To assess the clinical utility of the model, a decision curve analysis was performed. The decision curve was used to quantify the clinical utility of the model, displaying standardized net benefit against risk threshold probability. The clinical impact curve showed the number of judged high-risk patients for different threshold probabilities and the number of true positives. To analyze the relationship between POHL and those secondary outcomes, propensity score matching was performed to reduce baseline differences between groups. Kaplan–Meier curves and log rank test were used for survival analysis.

Statistical analyses were performed using SPSS (IBM SPSS Statistics 26.0, SPSS Inc., Chicago, IL) and R language (version 4.0.3, www.R-project.org/). All hypothesis tests were two-sided, with a significance level of *p* < 0.05.

## Results

### Demographic Characteristic

**Figure 1:**
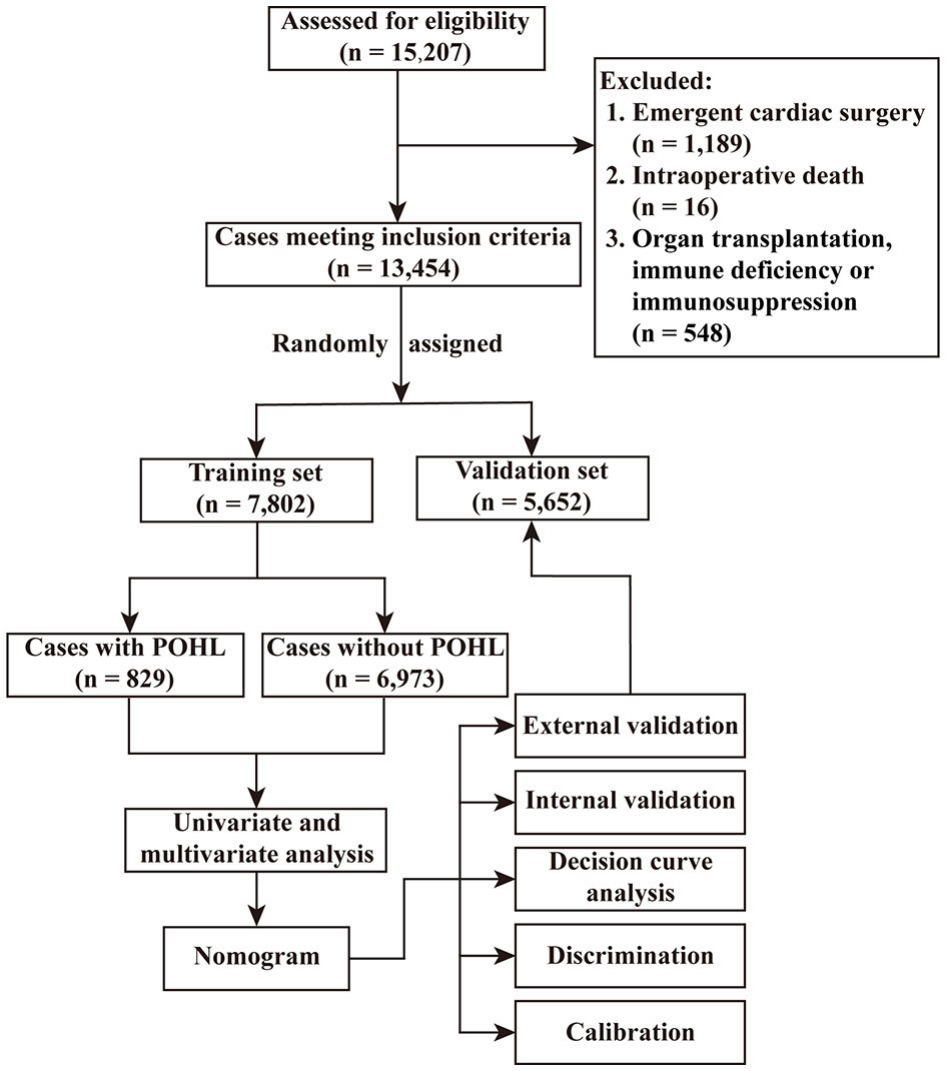
Flow chart of the study. POHL, postoperative hyperlactatemia.

Among the 15,207 adult patients who underwent cardiac surgery, 1,189 patients underwent emergency procedures, 548 patients experienced organ transplantation, immunosuppression, or immune deficiency, and 16 patients died intraoperatively. The remaining 13,454 patients fulfilling the inclusion criteria were randomly assigned to a training set (*n* = 7,802) and a validation set (*n* = 5,652), and were further analyzed (Figure 1). The average age of these patients was 51.59 ± 13.16 years and the proportion of men was 53.9%. The overall incidence of POHL after ECS was 10.6%. All the preoperative and intraoperative variables were well-balanced between the two sets (Table 1).

**Table 1:** Comparison of characteristics between the training and validation sets.

**Characteristic**	**Training set** ***n* = 7,802 (%)**	**Validation set** ***n* = 5,652 (%)**	***P*-value**
Demographics
Age (years)*^†^*	51.58 ± 13.15	51.60 ± 13.16	0.946
Male^§^	4,203 (53.9)	3,053 (54.0)	0.867
Body mass index (kg/m^2^)*^†^*	23.14 ± 3.29	23.10 ± 3.30	0.475
Smoking^§^	2,109 (27.0)	1,525 (27.0)	0.949
Drinking^§^	1,551 (19.9)	1,160 (20.5)	0.358
Underlying conditions			
Hypertension^§^	1,939 (24.9)	1,411 (25.0)	0.882
Diabetes mellitus^§^	594 (7.6)	465 (8.2)	0.192
Chronic obstructive pulmonary disease^§^	882 (11.3)	660 (11.7)	0.503
Cerebrovascular disease^§^	793 (17.5)	597 (18.3)	0.376
Peripheral vascular disease^§^	964 (21.3)	714 (21.8)	0.534
Renal insufficiency^§^	564 (7.2)	413 (7.3)	0.863
Gastrointestinal tract disease^§^	647 (8.3)	455 (8.1)	0.613
Atrial fibrillation^§^	1,542 (19.8)	1,066 (18.9)	0.191
General surgery history^§^	2,243 (28.7)	1,680 (29.7)	0.219
Cardiac surgery history^§^	537 (6.9)	392 (6.9)	0.905
New York Heart Association class III-IV^§^	1,342 (17.2)	946 (16.7)	0.480
Pulmonary artery hypertension^§^	2,278 (29.2)	1,642 (29.1)	0.854
Pericardial effusion^§^	1,005 (12.9)	746 (13.2)	0.589
Left ventricular ejection fraction (%)^*^	62 (57, 67)	62 (57, 67)	0.985
Laboratory values			
White blood cell count (×10^9^/L)^*^	5.7 (4.7, 6.8)	5.7 (4.7, 6.8)	0.397
Red blood cell count (×10^12^/L)^*^	4.3 (3.9, 4.7)	4.3 (3.9, 4.7)	0.687
Hemoglobin (g/l)^*^	130 (118, 141)	129 (118, 141)	0.579
Serum creatinine (μmol/L)^*^	71.3 (60.6, 84.0)	71.0 (60.3, 83.8)	0.364
Serum albumin (g/L)*^†^*	40.53 ± 3.83	40.64 ± 3.79	0.100
Serum globulin (g/L)*^†^*	24.66 ± 4.41	24.63 ± 4.35	0.653
Operative variables			
Cardiopulmonary bypass time (minutes)^*^	100 (77, 130)	100 (76, 128)	0.093
Aortic cross clamp time (minutes)^*^	67 (48, 89)	67 (47, 88)	0.147
Intraoperative transfusion of RBC (units)^*^	1 (0, 3)	1 (0, 3)	0.402

† *Normally distributed continuous variables are presented as means with standard deviations and analyzed by Student's t-test*.

* *Non-normally distributed continuous variables are presented as medians with interquartile ranges and analyzed by non-parametric test*.

§ *Categorical variables are presented as frequencies with percentages and analyzed by Chi-square test or Fisher's exact test. RBC, red blood cell*.

### Construction of a Full Nomogram for POHL

**Figure 2:**
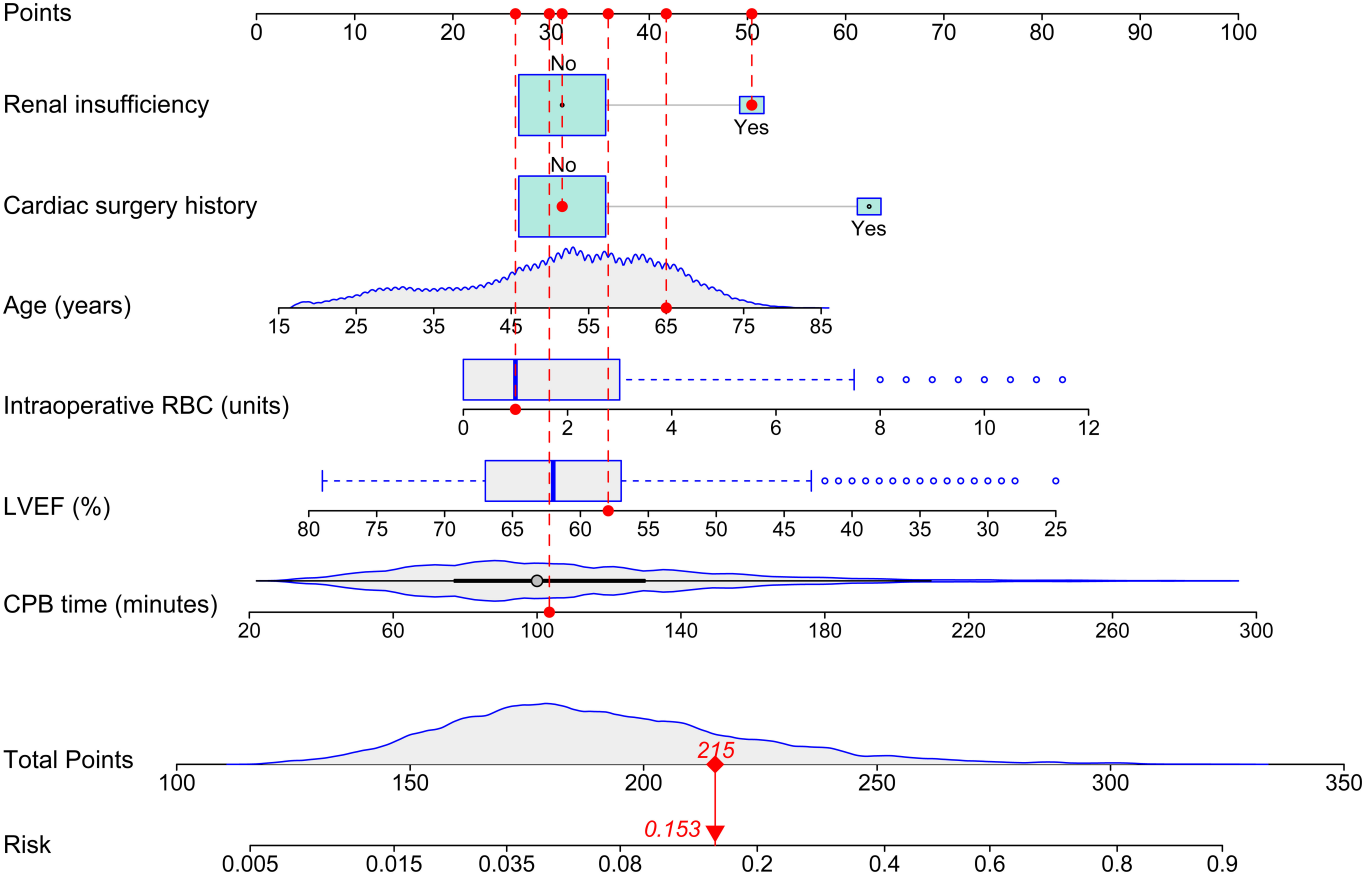
Nomogram for the prediction of postoperative hyperlactatemia after elective cardiac surgery. A true patient is shown to illustrate how to use the nomogram. This is a 65-year-old patient who has experienced his first-time cardiac surgery, with a history of renal insufficiency and an LVEF of 58%. The CPB time is 103 min and the volume of intraoperative RBC transfusion is one unit. The individual item score corresponding to each factor is presented at the top, and the total points are obtained from the sum of the scores corresponding to each factor by a red dot. Based on the given values of the six predictors, the patient can be intuitively mapped onto the nomogram. It can be clearly seen from the nomogram that the total points of this patient are 215 points and the corresponding probability of POHL is 0.153. CPB, cardiopulmonary bypass; LVEF, left ventricular ejection fraction; RBC, red blood cell.

A total of 829 patients developed POHL after ECS in the training set. The possible risk factors for POHL are displayed in Table 2. Collinearity diagnostics were performed before the multivariate model was constructed. By multivariate logistic regression analysis, six independent predictors associated with the development of POHL after ECS were identified, including age, LVEF, renal insufficiency, cardiac surgery history, the volume of intraoperative RBC transfusion, and CPB time (Table 3). Based on these independent predictors, a full nomogram was established to predict the risk of POHL after ECS (Figure 2). Regression coefficients of the variables were correspondingly converted to scores within a range of 0–100, reflecting their relative importance. The probability of POHL in a patient can be calculated easily by summing the points of all these predictors. A true case is presented in Figure 2.

**Table 2:** Univariate analysis of risk factors for POHL after elective cardiac surgery in the training set.

**Characteristic**	**Without POHL** ***n* = 6,973 (%)**	**With POHL** ***n* = 829 (%)**	**χ^2^/Z/t**	***P*-value**
Demographics			
Age (years)*^†^*	50.93 ± 13.16	57.02 ± 11.83	13.829	<0.001
Male^§^	3,698 (53.0)	505 (60.9)	18.531	<0.001
Body mass index (kg/m^2^)*^†^*	23.13 ± 3.31	23.17 ± 3.06	0.334	0.739
Smoking^§^	1,831 (26.3)	278 (33.5)	19.886	<0.001
Drinking^§^	1,345 (19.3)	206 (24.8)	14.383	<0.001
Underlying conditions
Hypertension^§^	1,660 (23.8)	279 (33.7)	38.482	<0.001
Diabetes mellitus^§^	539 (7.7)	55 (6.6)	1.264	0.261
Chronic obstructive pulmonary disease^§^	755 (10.8)	127 (15.3)	14.911	<0.001
Cerebrovascular disease^§^	1,200 (17.2)	190 (22.9)	16.498	<0.001
Peripheral vascular disease^§^	1,437 (20.6)	241 (29.1)	31.435	<0.001
Renal insufficiency^§^	412 (5.9)	152 (18.3)	170.610	<0.001
Gastrointestinal tract disease^§^	566 (8.1)	81 (9.8)	2.655	0.103
Atrial fibrillation^§^	1,308 (18.8)	234 (28.2)	41.889	<0.001
General surgery history^§^	2,037 (29.2)	206 (24.8)	6.887	0.009
Cardiac surgery history^§^	401 (5.8)	136 (16.4)	131.232	<0.001
New York Heart Association class III-IV^§^	1,136 (16.3)	206 (24.8)	38.100	<0.001
Pulmonary artery hypertension^§^	2,047 (29.4)	231 (27.9)	0.797	0.372
Pericardial effusion^§^	907 (13.0)	98 (11.8)	0.928	0.335
Left ventricular ejection fraction (%)^*^	62 (58, 67)	60 (53, 65)	11.102	<0.001
Laboratory values			
White blood cell count (×10^9^/L)^*^	5.7 (4.7, 6.8)	5.7 (4.9, 6.7)	0.775	0.438
Red blood cell count (×10^12^/L)^*^	4.3 (3.9, 4.7)	4.3 (4.0, 4.6)	0.574	0.566
Hemoglobin (g/l)^*^	130 (118, 141)	130 (119, 141)	0.260	0.795
Serum creatinine (μmol/L)^*^	70.9 (60.3, 83.5)	75.7 (63.4, 90.4)	6.781	<0.001
Serum albumin (g/L)*^†^*	40.58 ± 3.87	40.16 ± 3.52	3.191	0.001
Serum globulin (g/L)*^†^*	24.65 ± 4.37	24.77 ± 4.70	0.713	0.476
Operative variables			
Cardiopulmonary bypass time (minutes)^*^	97 (75, 126)	132 (102, 167)	19.953	<0.001
Aortic cross clamp time (minutes)^*^	66 (46, 87)	83 (63, 112)	15.107	<0.001
Intraoperative transfusion of RBC (units)^*^	1 (0, 2.5)	3 (1, 6)	17.825	<0.001

† *Normally distributed continuous variables are presented as means with standard deviations and analyzed by Student's t-test*.

* *Non-normally distributed continuous variables are presented as medians with interquartile ranges and analyzed by non-parametric test*.

§ *Categorical variables are presented as frequencies with percentages and analyzed by the chi-square test or Fisher's exact test. POHL, postoperative hyperlactatemia; RBC, red blood cell*.

**Table 3:** Multivariate analysis of independent risk factors for postoperative hyperlactatemia after elective cardiac surgery.

**Characteristic**	**Coefficient**	**Standard error**	**OR (95% CI)**	***P*-value**
Age (years)	0.028	0.004	1.029 (1.021–1.036)	<0.001
Renal insufficiency	0.695	0.117	2.003 (1.592–2.520)	<0.001
Cardiac surgery history	1.125	0.123	3.079 (2.418–3.922)	<0.001
Left ventricular ejection fraction (%)	−0.050	0.004	0.951 (0.943–0.960)	<0.001
Cardiopulmonary bypass time (minutes)	0.013	0.001	1.013 (1.011–1.015)	<0.001
Transfusion of red blood cell (units)	0.191	0.017	1.210 (1.170–1.252)	<0.001
Intercept	−2.921	0.346	0.054	<0.001

*CI, confidence interval; OR, odds ratio*.

### Validation and Assessment of the Full Nomogram

**Figure 3:**
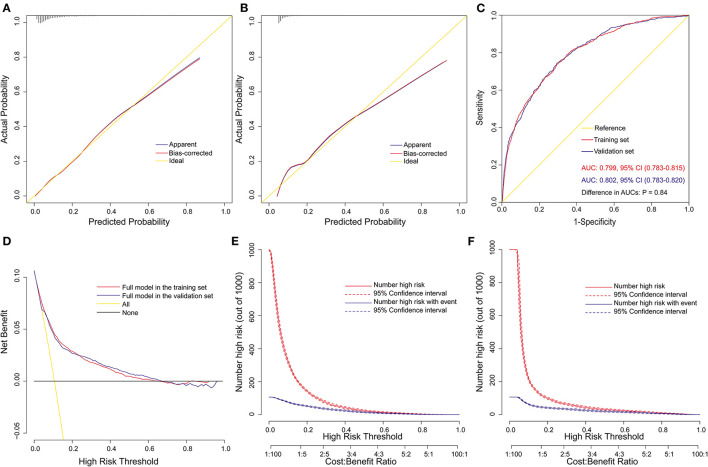
Assessment and validation of the full nomogram model. **(A)** calibration plot in the training set; **(B)** calibration plot in the validation set; **(C)** ROC curves in both the training and validation sets; **(D)** decision curves in both the training and validation sets; **(E)** clinical impact curve in the training set; and **(F)** clinical impact curve in the validation set. AUC, area under the receiver operating characteristic curve; CI, confidence interval; ROC curve, receiver operating characteristic curve.

The full nomogram was validated by using both internal and external validation. The calibration was tested by both visual inspections of the calibration plots and the goodness-of-fit test. The full nomogram was well-calibrated in both the training (Hosmer–Lemeshow χ^2^ = 3.72, *p* = 0.881) and the validation (Hosmer–Lemeshow χ^2^ = 6.86, *p* = 0.551) sets. By visual inspection, the full nomogram also showed good calibration (Figures 3A,B). To evaluate the predictive performance of the full nomogram, the ROC curves were drawn in both sets (Figure 3C). The AUC was 0.799 (95% CI, 0.783–0.815) in the training set and 0.802 (95% CI, 0.783–0.820) in the validation set, respectively. No significant difference was found between the two AUCs (*p* = 0.838). To assess the clinical utility, a decision curve analysis was performed. Compared with “no intervention” or “intervention for all” strategies, the use of the full nomogram could obtain more clinical net benefits when the risk threshold was between 0.07 and 0.61 (Figure 3D). The clinical impact curves also showed that the number of deemed high risk to develop POHL and the number of patients with actual POHL events tended to be close within this risk threshold, indicating prominent predictive power, and good clinical utility (Figures 3E,F).

### Establishment, Validation, and Assessment of a Preoperative Prediction Model

**Figure 4:**
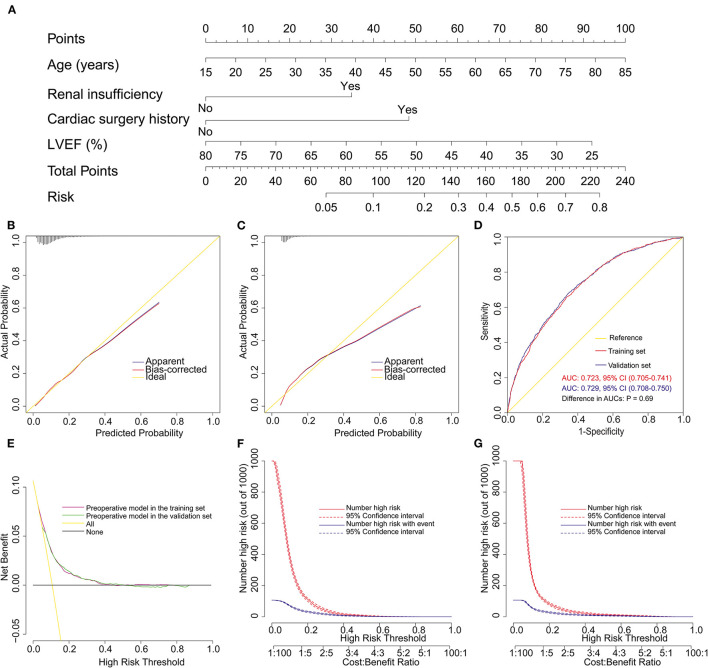
Development, assessment, and validation of the preoperative nomogram model. **(A)** Preoperative nomogram predicting the development of postoperative hyperlactatemia in patients undergoing elective cardiac surgery; **(B)** calibration plot in the training set; **(C)** calibration plot in the validation set; **(D)** ROC curves in both the training and validation sets; **(E)** decision curves in both the training and validation sets; **(F)** clinical impact curve in the training set; **(G)** clinical impact curve in the validation set. AUC, area under the receiver operating characteristic curve; CI, confidence interval; LVEF, left ventricular ejection fraction; ROC curve, receiver operating characteristic curve.

To facilitate clinical application, a second nomogram model was established using only preoperative variables. Age, LVEF, renal insufficiency, and cardiac surgery history were identified as independent risk factors for POHL in patients undergoing ECS by multivariate logistic regression analysis (Table 4). A preoperative nomogram model was then constructed based on these four preoperative predictors (Figure 4A). This model was also well-calibrated in the training (Hosmer-Lemeshow χ^2^ = 4.16, *p* = 0.842) and validation (Hosmer-Lemeshow χ^2^ = 7.11, *p* = 0.525) sets (Figures 4B,C). The AUCs were, respectively, 0.723 (95% CI, 0.705–0.741) and 0.729 (95% CI, 0.708–0.750) in the training and validation sets (Figure 4D), and there was no significant difference between the two AUCs (*p* = 0.688). More clinical net benefits could be obtained using this nomogram when the risk threshold was between 0.08 and 0.42, and it also showed good predictive power and clinical utility (Figures 4E–G).

**Table 4:** Multivariate analysis of preoperative predictors for postoperative hyperlactatemia after elective cardiac surgery.

**Characteristic**	**Coefficient**	**Standard error**	**OR (95% CI)**	***P*-value**
Age (years)	0.040	0.003	1.040 (1.033–1.047)	<0.001
Renal insufficiency	0.963	0.109	2.620 (2.115–3.244)	<0.001
Cardiac surgery history	1.340	0.114	3.821 (3.057–4.776)	<0.001
Left ventricular ejection fraction (%)	−0.046	0.004	0.955 (0.947–0.963)	<0.001
Intercept	−1.737	0.313	0.176	<0.001

*CI, confidence interval; OR, odds ratio*.

### Risk Intervals of POHL

**Figure 5:**
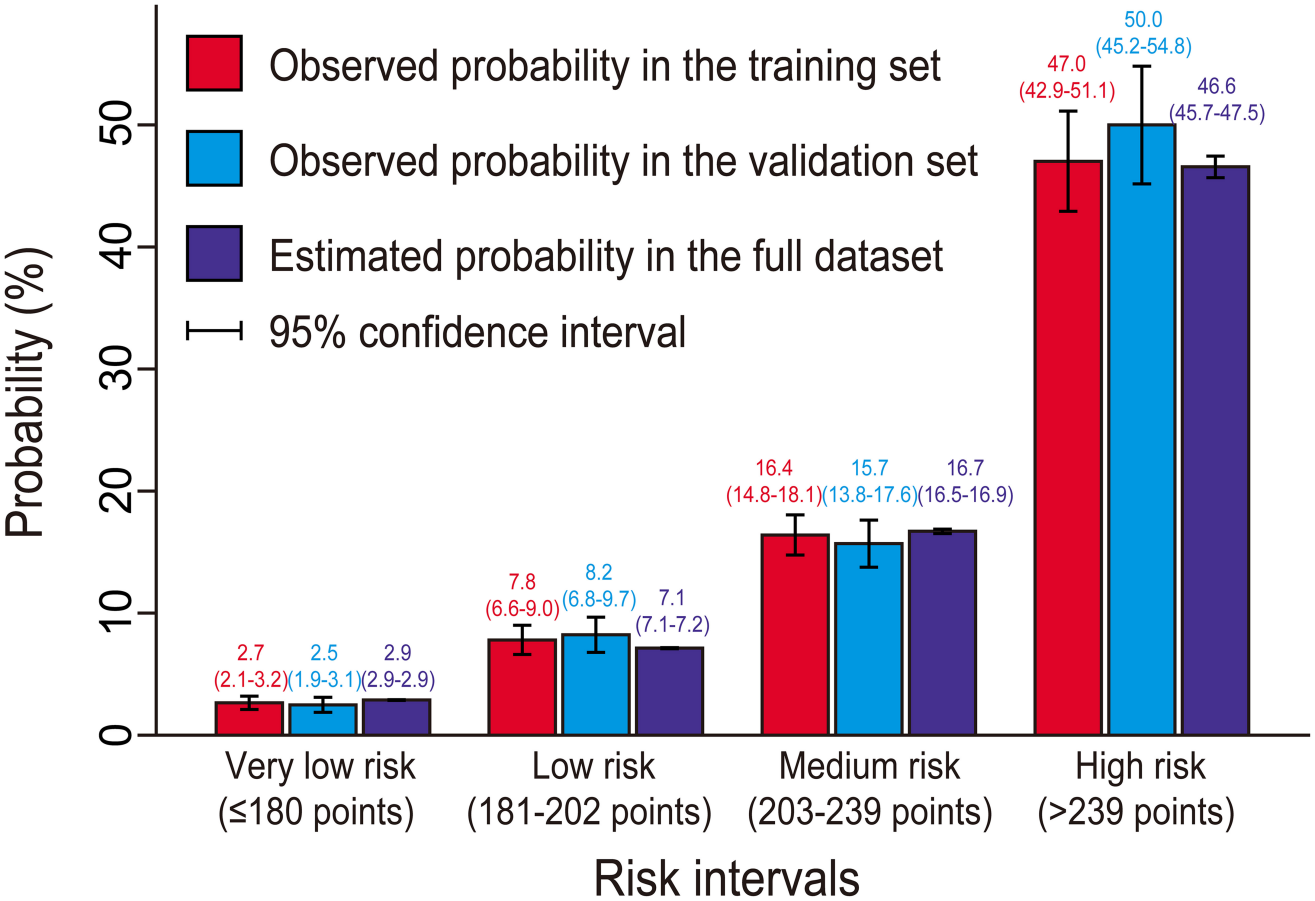
Observed and predicted probabilities of postoperative hyperlactatemia by risk intervals. No significant difference was found among the predicted probabilities and the observed probabilities in the training and validation sets in the same risk interval (*p* > 0.05), indicating good agreement. However, significant differences were found among different risk intervals (*p* <0.001), indicating good classification.

Based on the full nomogram model and clinical practice, we stratified the study population into four-risk intervals, named very low risk, low risk, medium risk, and high risk groups (Table 5). The estimated probabilities of <5% were considered as very low-probability events and thus we chose 5% as a cutoff value to divide the very low risk and low risk groups. The estimated probabilities of between 5 and 10% were considered as low risk, 10–30% as medium risk, and >30% as high risk, respectively. The corresponding cutoff values on the nomogram were, respectively, ≤ 180, 181–202, 203–239, and >239 points. In this study, 43.1% of the patients were classified into the very low risk group, 24.9% into the low risk group, 24.7% into the medium risk group, and only 7.3% into the high risk group. Estimated probabilities and observed probabilities in the training and validation sets of the four risk intervals are presented in Figure 5, indicating good consistency in the same risk interval and significant differences among different risk intervals.

**Table 5:** Risk intervals of postoperative hyperlactatemia based on the full nomogram model.

**Risk intervals**	**Very low risk** **(≤180 points)**	**Low risk** **(181–202 points)**	**Medium risk** **(203–239 points)**	**High risk** **(>239 points)**
Estimated probability (%)	<5	5–10	10–30	>30
Observed probability, % (95% CI)	2.6 (2.2–3.0)	8.0 (7.1–8.9)	16.1 (14.9–17.4)	48.3 (45.2–51.4)
No. of patients (%)	5,792 (43.1)	3,354 (24.9)	3,320 (24.7)	988 (7.3)

*CI, confidence interval*.

### Outcomes

**Figure 6:**
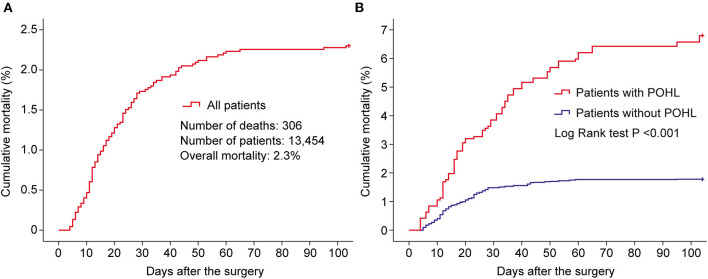
Kaplan–Meier curves showing the cumulative mortality of all the patients who underwent elective cardiac surgery **(A)**, and the comparison of cumulative mortality in patients with and without POHL **(B)**. POHL, postoperative hyperlactatemia.

Postoperative hyperlactatemia after ECS developed in 1,430 of the 13,454 patients (10.6%). By univariate analysis, we observed significantly poorer outcomes in patients with POHL compared with patients without that (Table 6). A total of 306 patients (2.3%) died in our results; however, the mortality was significantly higher in patients with POHL (Figure 6). To identify whether POHL was independently associated with a higher risk of readmission to ICU and mortality, we further conducted multivariate logistic regression analysis. We found that POHL was an independent predictor for both of the two outcomes, and patients with POHL had a 2.13-fold increased risk of readmission to ICU (*p* < 0.001) and a 2.34-fold increased risk of mortality (*p* < 0.001). To evaluate the influence of POHL on the lengths of ICU and hospital stay, we further performed propensity score matching by nearest-neighbor matching without replacement with an algorithm of 1:1 matching, yielding 1,350 matched pairs of patients. In this study population, the length of ICU stay in patients with and without POHL was, respectively, 4 (3, 7) and 4 (2, 6) days (Z = 7.082, *p* < 0.001) and the length of hospital stay in patients with and without POHL was, respectively, 17 (13, 23) and 16 (11, 20) days (Z = 6.258, *p* < 0.001).

**Table 6:** Outcomes in patients with and without POHL after elective cardiac surgery.

**Outcome**	**All patients** ***n* = 13,454 (%)**	**Without POHL** ***n* = 12,024 (%)**	**With POHL** ***n* = 1,430 (%)**	**χ^2^/Z**	***P*-value**
Readmission to ICU	440 (3.3)	314 (2.6)	126 (8.8)	155.282	<0.001
ICU stay (days)	3 (2, 4)	3 (2, 4)	5 (3, 7)	29.783	<0.001
Hospital stay (days)	14 (11, 18)	14 (10, 18)	18 (13, 24)	21.953	<0.001
Mortality	306 (2.3)	212 (1.8)	94 (6.6)	133.045	<0.001

*ICU, intensive care unit; POHL, postoperative hyperlactatemia*.

## Discussion

The elevated level of postoperative lactate has been well-recognized to be associated with poor outcomes (1, 10, 14), which was confirmed again by the results of this study. The incidence of POHL after ECS was 10.6%, close to previous reports (6, 7). The overall mortality was 2.3%, similar to previous studies (14). Nonetheless, the mortality was significantly higher in patients with POHL compared with patients without that. Moreover, significantly higher probabilities of other poor clinical outcomes such as readmission to ICU and the length of hospital stay were also observed in those with POHL. However, the optimal management of patients with POHL is little known (17), emphasizing the importance of early management of high risk populations that may develop POHL.

To our knowledge, our study represents the first large-scale study that focuses on the risk factors for POHL after ECS and the establishment of nomogram models globally. In this study, we developed and validated two nomogram models for POHL after ECS using data from 13,454 patients in four institutions. By univariate and multivariate analysis, we identified four preoperative and two intraoperative independent risk factors for POHL. A full nomogram model based on all the six predictors and a preoperative nomogram model based on the four preoperative predictors were then established. Both models indicated good calibration, discrimination, and clinical usefulness. Finally, on the basis of the full nomogram model and clinical practice, we defined four risk intervals to facilitate clinical application.

It is well-known that lactate is produced by pyruvate during glycolysis, and its increase comes from an increase in production and a decrease in clearance. However, the parts that contribute more to the development of hyperlactatemia vary greatly from person to person (15, 18). In this study population, age, renal insufficiency, LVEF, cardiac surgery history, CPB time, and RBC transfusion were identified as independent predictors for POHL. Indeed, these factors were also found to be independently associated with renal impairment and pulmonary dysfunction after cardiac operations (19, 20). Thus, we speculate that POHL may act as an abnormal intermediate product and a causal link between clinical features and outcomes.

The history of previous cardiac surgery was identified as the most significant predictor for POHL after ECS in our analysis. Reentry procedures in redo cardiac surgery are technically demanding and carry elevated operative risk due to anatomical planes loss, injuries to numerous structures, and hemorrhage (21–23). Bianco et al. found that more blood product transfusion was required in reoperative patients, and speculated that transfusion requirements might be related to injuries upon reentry and prolonged operative and CPB time (24). In addition, patients undergoing reoperation often had older age and more comorbidities (24, 25). In this study, age was positively correlated to the peak lactate value. However, the results with regard to the correlation between age and elevated lactate were mixed in the literature, which may be caused by different measurement time points of lactate (10, 11).

Our results showed that the lower the preoperative LVEF, the higher risk of developing POHL, which was consistent with previous studies (8). It is well-known that low LVEF can affect tissue perfusion. Myocardial stunning caused by ischemia-reperfusion states, as a frequent consequence following CPB, may lead to decreased left ventricular function and thus can further aggravate the accumulation of lactate (26). In this study, renal insufficiency was another independent preoperative risk factor. This may be due to the fact that the kidney mostly relies on lactate as the gluconeogenic substrate, and renal insufficiency could affect the systemic clearance of lactate, giving rise to hyperlactatemia (27). In addition, patients undergoing chronic hemodialysis are more likely to be deficient in thiamine due to strict dietary control, which may cause pyruvate to shift to anaerobic metabolism (28).

The preoperative nomogram model comprising four preoperative factors mentioned above was moderately accurate in predicting POHL after ECS. These factors are simple and easily accessible in clinical practice. This nomogram allows patients to intuitively and easily understand their conditions. For clinicians, this may help to evaluate the risk of patients and formulate feasible plans.

Although this study was conducted in patients undergoing ECS, as expected, the independent association between CPB duration and POHL development was still significant, in line with previous studies (3, 10). Evans et al. reported that the duration of CPB longer than 3 h was associated with 5.8-fold increased odds of POHL (13). The stress of cardiac surgery has been demonstrated to cause decreased pyruvate dehydrogenase quantity and activity and depletion of thiamine levels, resulting in hyperlactatemia (29, 30). Regrettably, Luger et al. found that the intravenous thiamine supplementation prior to cardiac surgery failed to significantly reduce postoperative blood lactate concentration (31). In addition, Mustafa et al. indicated that the CPB procedure decreased lactate clearance, which was possibly linked to the liver dysfunction in elective surgery (32). Moreover, microvascular thrombosis was found to be linked to cardiac surgery with CPB, and insufficient oxygen supply in the microcirculation area increased lactate levels (9). Without a doubt, the extension of CPB time will exacerbate this situation.

The loss of clotting factors and platelets induced by CPB leads to coagulation dysfunction, which may relate to an increased risk of bleeding (33). To some extent, more blood transfusion requirements always imply more blood loss during the operation. Moreover, hemodilution as a necessary part of the CPB process may further increase the possibility of demand for blood transfusion. In this study, the volume of RBC transfusion was also identified as an independent risk factor for POHL. It is undeniable that increasing oxygen delivery is the physiologic benefit of blood transfusion, and several studies have shown that RBC transfusion can improve tissue oxygenation and increase functional capillary density in the microcirculation (34, 35). However, increasing evidence suggested that massive transfusion of RBC increased the risk of adverse outcomes in cardiac surgical patients (36–38). Surgenor et al. indicated that RBC transfusion initiated a systemic inflammatory response, leading to local microvascular occlusion and tissue hypoxia (38). They also found that exposure to RBC transfusion during surgery was associated with low-output heart failure, which may have a relationship with hyperlactatemia (38). The results of randomized controlled trials also indicated that restrictive RBC transfusion strategy was safe in both adults and pediatric patients who underwent cardiac surgery, and it had obvious resources and economic advantages (39, 40).

In addition, limiting the effect of hemodilution on renal function was effective in reducing acute kidney injury rates (41). We speculate that it is reasonable to limit hemodilution and thus decrease transfusion requirements, which may reduce lactate levels due to the fact that renal injury may affect lactate metabolism. Of course, correction of preoperative anemia was also a feasible option for patients undergoing elective surgery to reduce intraoperative blood transfusion (42). Minimized CPB has also been reported to have beneficial effects on decreasing transfusion rate (43), which may be due to lower inflammatory response (44). Undeniably, skilled surgical operation with mature teamwork also plays an important part to reduce CPB and surgery time.

The full nomogram model involved four preoperative and two intraoperative predictors, showing a good prediction efficiency and clinical utility, and was well-validated in the independent cohort. Its character as a graphical prediction tool makes clinicians convenient to do a risk assessment and clinical intervention considerations. Moreover, the utilization of our models may help reduce the significant increase of lactate levels after ECS and, thus, may have a positive effect on reducing postoperative adverse outcomes.

Several measures have been reported to have an effect on alleviating POHL, such as minimal volume ventilation in robotically assisted cardiac surgery, continuous ultrafiltration through polyethersulfone membrane during CPB, and modified glucose–insulin–potassium regimen (45–47). There may exist a huge waste of labor and material resources if these measures are applied to all patients without selection, especially to those who are at very low risk. However, implementing these auxiliary interventions in patients who are identified as high risk by our risk models may be more effective and more cost-efficient for clinical application.

There are some limitations in this study that deserve mention. First, this was a retrospective observational study, and POHL was diagnosed on the basis of medical records. Despite having carefully reviewed all the records, we cannot ensure that all patients who developed POHL were included. Second, some possible risk factors for POHL after ECS were not available in this study, such as intraoperative hemodynamic data and the use of positive inotropic drugs. Nevertheless, the nomogram models performed well in discrimination, calibration, and clinical utility. Third, the records of POHL were limited within the first postoperative 12 h, which may underestimate the true incidence. However, this was in line with the main purpose of this study, as previous studies have indicated that early POHL was significantly associated with poorer prognosis.

## Conclusion

In this study, we developed and validated two nomogram models to assess the probability of POHL in patients undergoing ECS. The full nomogram model incorporated both preoperative and intraoperative variables, and the preoperative nomogram model used only preoperative variables. Both nomogram models demonstrated good discrimination, calibration, and clinical utility. Based on the full nomogram model, four risk intervals were divided for better clinical practice. Risk assessment and targeted interventions based on the predictive models may be conducive to decreasing the incidence of POHL in patients undergoing ECS.

## Data Availability Statement

The raw data supporting the conclusions of this article will be made available by the authors, without undue reservation.

## Ethics Statement

The studies involving human participants were reviewed and approved by the Ethical Committee of the Union Hospital (No. 0521), Tongji Medical College, Huazhong University of Science and Technology, Wuhan, China. Written informed consent for participation was not required for this study in accordance with the national legislation and the institutional requirements.

## Author Contributions

DW, XH, AZ, and XD conceptualized and supervised the study. DW, JW, SL, FX, XL, AZ, and HW collected the clinical data. DW, SW, JW, SL, FX, XL, HW, and XH summarized all the data. DW, SW, JW, and SL drafted the manuscript. DW, SW, XH, AZ, and XD revised the final manuscript. All authors read and approved the final manuscript.

## Funding

This study was supported by the National Natural Science Foundation of China (Grant nos. 81800413 and 81801586).

## Conflict of Interest

The authors declare that the research was conducted in the absence of any commercial or financial relationships that could be construed as a potential conflict of interest.

## Publisher's Note

All claims expressed in this article are solely those of the authors and do not necessarily represent those of their affiliated organizations, or those of the publisher, the editors and the reviewers. Any product that may be evaluated in this article, or claim that may be made by its manufacturer, is not guaranteed or endorsed by the publisher.

## Abbreviations

AUC: area under the receiver operating characteristic curve
CI: confidence interval
CPB: cardiopulmonary bypass
ECS: elective cardiac surgery
ICU: intensive care unit
LVEF: left ventricular ejection fraction
POHL: postoperative hyperlactatemia
RBC: red blood cell
ROC: receiver operating characteristic.
